# Comparative Evaluation of LAMP, qPCR, Conventional PCR, and ELISA to Detect Ralstonia solanacearum in Kenyan Potato Fields

**DOI:** 10.1094/PDIS-03-18-0489-RE

**Published:** 2019-03-20

**Authors:** Lilian A. Okiro, Matthew A. Tancos, Steven G. Nyanjom, Christine D. Smart, Monica L. Parker

**Affiliations:** 1 Department of Biochemistry and Molecular Biology, Egerton University, Njoro Campus, PO Box, 536 – 20115, Egerton, Kenya; 2 Department of Biochemistry, Jomo Kenyatta University of Agriculture and Technology, Nairobi, Kenya; 3 Biosciences Eastern and Central Africa–International Livestock Research Institute (BecA-ILRI) Hub, Nairobi, 00100, Kenya; 4 Plant Pathology and Plant-Microbe Biology Section, School of Integrative Plant Science, Cornell University, Geneva, NY, U.S.A.; and; 5 CGIAR Research Program on Roots, Tubers and Bananas, International Potato Center, Nairobi, Kenya

## Abstract

Bacterial wilt caused by *Ralstonia solanacearum* is considered among the most damaging diseases of potato in Sub-Saharan Africa and the most significant biotic constraint of potato production alongside late blight. Unlike late blight,which can be managed by chemical means, *R. solanacearum* can only be managed through cultural methods and clean seed. Laboratory testing to certify seed before planting is required to confirm the absence of the pathogen in Kenya. A loop-mediated isothermal amplification (LAMP) assaywas developed using the UDP-(3-O-acyl)-*N*-acetylglucosamine deacetylase gene (*IpxC*) to screen seed potato for *R. solanacearum* strains. The assay was assessed using DNA extracted from *R. solanacearum* and other soil and potato pathogens to demonstrate specificity and sensitivity. The LAMP assay was validated using field samples from different potato growing regions of Kenya collected over two growing seasons and compared with established nucleic acid and protein-based assays. The *IpxC* LAMP assay was found to be specific and sensitive to *R. solanacearum*, detecting as low as 2.5 pg/ml of R. solanacearum DNA. Of the 47 potentially infected field samples collected, both *IpxC* LAMP and quantitative polymerase chain reaction (PCR) detected *R. solanacearum* DNA in 90% of the samples, followed by conventional PCR (86%) and ELISA (75%). This *IpxC* LAMP assay is a promising diagnostic tool to rapidly screen for *R. solanacearum* in seed potato with high sensitivity in Kenya

*Ralstonia solanacearum* is a bacterial pathogen that causes disease in greater than 450 monocot and dicot plant species (Guidot et al. 2009; Hayward 1991; N’Guessan et al. 2012; Stulberg et al. 2015; Weller et al. 2000). In Ethiopia and Kenya, the pathogen has been reported to cause losses of 30 to 70% at altitudes of 1,560 to 3,300 and 1,800 to 2,800 m above sea level, respectively (Sharma, personal communication; Wagura et al. 2011). The disease is a serious issue for potato production in Kenya, with one study revealing 77% of potato farms were affected by *R. solanacearum*, followed by late blight at 67% and viral diseases at 12% (Gildemacher et al. 2009). The pathogen spreads through contaminated planting material, soil, water run-off,and farm implements. Vegetatively propagated crops, such as potato, are particularly vulnerable to infection because of the inadvertent spreading of the pathogen owing to latent infection. The use of clean planting material and good cultural practices are the most effective methods to manage the pathogen (Hayward 1991).

In developing countries, underdeveloped potato seed systems have resulted in farmers visually selecting and saving seed from harvested potato tubers and reusing the same tubers in subsequent seasons. *R. solanacearum* has spread under such circumstances, because farmers tend to retain small unmarketable tubers for seed, which are often infected with the pathogen (Demo et al. 2015). Tubers originating from latently infected plants further compound the problem, because visual inspection in the field cannot identify infected tubers.

There are opportunities for rapid and specific diagnostic methods that are sufficiently sensitive to detect latent R. solanacearum populations in bulk tuber samples and remain in compliance with the phytosanitary regulations. An array of techniques has been developed detect *R. solanacearum* including semiselective culture techniques (Elphinstone 1996), serological assays such as enzyme-linked immunosorbent assay (ELISA) (Priou et al. 1997, 2006), and nucleic acid amplification-based assays using polymerase chain reaction (PCR) (Chen et al. 2010; Dittapongpitch and Surat 2003; Stulberg et 2015; Weller et al. 2000). Isothermal amplification methods, such as loop-mediated isothermal amplification (LAMP) assays, have the potential to overcome some of the cost barriers associated with the aforementioned techniques by facilitating DNA amplification without expensive thermocycling equipment (Kubota and Jenkins 2015; Kubota et al. 2008).

LAMP amplifies targeted nucleic acid sequences and has been used to detect various plant, human, and animal pathogens (Gosch et al. 2012; Kubota and Jenkins 2015; Mitarai et al. 2011; Obura et al. 2011; Tomlinson et al. 2013; Wastling et al. 2010; Yasuhara-Bell et al. 2013). The technique uses a set of six primers recognizing different regions of the targeted DNA (Mori et al. 2001), hence enhancing specificity. LAMP reactions are rapid and efficient, producing visible amplification products in 30 to 60 min (Notomi et al. 2000). The quantity of amplification product allows simple endpoint formats to determine positive and negative samples (Wastling et al. 2010). LAMP results can be determined in a variety of ways including the use of real-time turbidimetry and turbidity (Kubota and Jenkins 2015; Mori et al. 2001) and gel electrophoresis (Notomi et al. 2000).

Several colorimetric methods to interpret results within the reaction tube have been developed, including the use of DNA intercalating dyes (Tomlinson et al. 2007) and metal ion indicator methods (Goto et al. 2009; Ravindran et al. 2012; Tomita et al. 2008; Tomlinson et al. 2010a,b). Metal ion indicator methods, such as hydroxynaphthol blue, have provided the simplest approach, because they are added together with the other reagents before incubation so that amplification and detection are combined in a single processing step. The hydroxynaphthol blue method is a closed tube system that minimizes contamination and allows the color changes to be assessed with the naked eye, thereby reducing the cost of a detection system. This enables the technique to be applied in laboratories with limited facilities or in the field (Goto et al. 2009; Ravindran et al. 2012).

The standard method of testing seed potato in Kenya for *R. solanacearum* relies on antibody-based assays, such as ELISA. The nitrocellulose membrane ELISA (NCM ELISA) method takes up to 4 days from sampling to detection because of an enrichment step involving culturing the test sample on semiselective broth (Elphinstone 1996) for 48 h prior to ELISA detection.

Nucleic acid-based assays to detect *R. solanacearum* have been previously developed to target several genes including 16S rRNA, endoglucanase (*egl*), and a flagellar subunit (*fliC*); however, these studies used expensive quantitative PCR (qPCR) thermocyclers (Kubota and Jenkins 2015; Kubota et al. 2008; Lenarčič et al. 2014; Stulberg et al. 2016). The use of expensive equipment limits adaptability of the technology, particularly in developing countries. Various colorimetric LAMP assays have been reported, such as turbidity and use of intercalating dyes (Kubota et al. 2008; Tomlinson et al. 2007). However, these methods have increased the risk of contamination because the dyes are added post-LAMP reaction, which requires opening reaction tubes. To better manage the bacterial wilt pathogen, a faster and cheaper method that can easily be adapted is in demand.

This manuscript describes the development of a *R. solanacearumspecific* LAMP assay that targets the UDP-(3-*O*-acyl)-*N*-acetylglucosamine deacetylase (*IpxC*) gene and uses hydroxynaphthol blue for quick and reliable endpoint visualization. The assay involves minimal resources and a simple visualization format that can be used in Sub-Saharan African labs or official points of entry to screen potato tubers for *R. solanacearum*.

## Materials and Methods

**LAMP assay development.** *Primer design*. Sequences targeting different genes of *R. solanacearum* genome were obtained from NCBI database (https://www.ncbi.nlm.nih.gov/genbank/). Two software programs, PrimerExplorer V4 (Eiken Co., Tokyo, Japan) and LAMP Designer (PREMIER Biosoft, Palo Alto, CA), were used to develop novel *R. solanacearum*-specific primers. Four genes previously shown to be specific to *R. solanacearum* were targeted for primer design, which included the *egl* (Huang et al. 2009), *fliC* (Kubota et al. 2008), 16S rRNA (Weller et al. 2000), and the upstream region of the *IpxC* gene (Chen et al. 2010). Of the four *R. solanacearum* genes, *IpxC* was selected for further analyses. Sequences targeting the *IpxC* gene of *R. solanacearum* (GenBank accession AF254622) were used to design the six *IpxC* primer sequences (Table 1). The *IpxC* primer sequences were analyzed by basic local alignment search tool (BLAST) and confirmed to target approximately 540 to 760 bp regions of the *R. solanacearum IpxC* gene.

**Table 1:** Loop-mediated isothermal amplification (IpxC LAMP), quantitative polymerase chain reaction (PCR), and conventional PCR primers used to detect *Ralstonia solanacearum* in potato

Primer	Sequence (59-39)	Target	Reference
IpxC	F3: GCTACACCCGCGAAATCG	UDP-3-O-acyl-GlcNAc deacetylase	AF254622^a^
	B3: AGCGGATAGCCGACCAC		
	FIP: ACGATCGCGTTGTCCAGGCTGCACCTTCGGCTTTGCCCA		
	BIP: ACGAGCACCGCATGCTGAACGCGTCCAGAATCTTGTGG		
	LF: TCCCGCAGCATCTCGACCTC		
	LB: CGATGAACTGCGCTATGGC		
RSF	GTGCCTGCCTCCAAAACGACT	Upstream region of the UDP-3-O-acyl-GlcNAc deacetylase	Chen et al. (2010)
RSR	GACGCCACCCGCATCCCTC		
759	GTCGCCGTCAACTCACTTTCC	ACH0732	Opina et al. (1997)
760	GTCGCCGTCAGCAATGCGGAATCG		

a GenBank accession number.

*Bacterial DNA preparation.* The *R. solanacearum* strains, representing all four phylotypes, and nontarget strains were used to evaluate primer specificity (Tables 1 and 2). *R. solanacearum* strains were grown aerobically on semiselective agar (Elphinstone 1996) and/or medium composed of casamino acid, peptone, and glucose at 30°C for 24 to 72 h. A single colony from each *R. solanacearum* strain was harvested and used for DNA extraction, and some were stored on beads (Copan Diagnostics, Murrieta, CA) at –70°C. Other bacterial strains were grown in nutrient broth with constant shaking and/or on nutrient agar at 30°C for 24 to 48 h.

Purified genomic DNA from *R. solanacearum*, potato bacterial pathogens, and nonpathogenic environmental bacterial strains (Table 2) were extracted with the method of Chen and Kuo (1993) and the commercial kits ZR Plant/Seed DNA MiniPrep (Zymo Research Corp., Irvine, CA) and PureLink Genomic DNA Mini Kit (Africa Biosystems, Nairobi, Kenya), according to the manufacturers’ instructions. Genomic DNA was quantified with the Nano-Drop ND-1000 (NanoDrop, Wilmington, DE).

**Table 2:** Bacterial strains used in this study and tested by IpxC loop-mediated isothermal amplification^a^

Species	Strain	Phylotype	Initial host	*IpxC*	Source or reference^b^
*Ralstonia species complex strains*					
*Ralstonia solanacearum*	J	IIA	Blueberry	√	C. Allen, U.S.A.
	UW551	IIB	Geranium	√	C. Allen, U.S.A.
	GMI1000	I	Tomato	√	P. Prior, France
	IPO1690	IIB	–	√	P. Prior, France
	CMR15	III	–	√	P. Prior, France
	PSI07	IV	–	√	P. Prior, France
	ACH0732	–	–	√	P. Prior, France
	ATCC 11696	–	–	√	ATCC
	ATCC BAA-1115	–	–	√	ATCC
*Ralstonia syzygii* subsp. *indonesiensis*	DMSZ 27480	IV	–	√	DMSZ
*Ralstonia syzygii* subsp. *celebesensis* BDB	DMSZ 27477	IV	–	x	DMSZ
Non-*R. solanacearum* species complex					
*Ralstonia pickettii*	DMSZ 6297	–	–	x	DMSZ
*Ralstonia mannitolilytica*	DMSZ 17512	–	–	x	DMSZ
*Burkholderia plantarii*	DMSZ 7128	–	–	x	DMSZ
*Burkholderia cepacian*	DMSZ 7288	–	–	x	DMSZ
*Burkholderia thailandensis*	DMSZ 13276	–	–	x	DMSZ
*Burkholderia pyrrocinia*	DMSZ 10685	–	–	x	DMSZ
*Cupriavidus necator*	DMSZ 15444	–	–	x	DMSZ
*Pectobacterium atrosepticum (Erwinia carotovora* subsp. *atroseptica)*	CU3351	–	Potato	x	Cornell Univ., U.S.A.
*Pectobacterium carotovorum subsp*. carotovorum (*Erwinia carotovora* subsp. *carotovora)*	71	–	Potato	x	Murata et al. (1990)
*Pectobacterium carotovorum* subsp. *carotovorum*	ATCC 15713	–	Potato	x	Cornell Univ., U.S.A.
*Agrobacterium vitis*	CG49	–	Grape	x	Ophel and Kerr (1990)
*Clavibacter michiganensis* subsp. *michiganensis*	0651	–	Tomato	x	Cornell Univ., U.S.A.
*Pseudomonas fluorescens*	09110	–	Environment	x	Cornell Univ., U.S.A.
*Pseudomonas fulva*	0430	–	Environment	x	Cornell Univ., U.S.A.
*Bacillus thuringiensis*	361	–	Soil	x	Cornell Univ., U.S.A.
*Streptomyces spp.*	83-01-03	–	Potato	x	Cornell Univ., U.S.A.
Potato tuber	–	–	Potato	x	International Potato Center, Kenya

a Symbols: – = phylotype and host unknown;

√ = positive test result for bacterial strains tested; and x = negative test result for bacterial strains tested.

b ATTC = American Type Culture Collection, Manassas, VA, and DMSZ = German Collection of Microorganisms and Cell Cultures, Braunschweig, Germany.

*LAMP assay*. LAMP reactions were performed in 25-F020µl volume containing 0.2 µM each of external primers (F3 and B3), 2.0 µM each of internal primers (FIP and BIP), 0.8 µM each of loop primers (LF and LB) (Integrated DNA Technologies, Coralville, IA), 0.8 M betaine (Sigma-Aldrich, Munich, Germany), 120 µM of hydroxynaphthol blue (Sigma-Aldrich), 1.40 mM of each dNTP (New England Biolabs, Ipswich, MA), 6 mM of MgSO_4_, 1× isothermal DNA buffer (New England Biolabs), 8 U of *Bacillus stearothermophilus* DNA polymerase (New England Biolabs), and 2 µl of bacterial DNA (10 to 20 ng/µl) (Wastling et al. 2010) (Table 1). All reagents were added preincubation, and the LAMP reaction was carried out at 65°C for 1 h, terminated at 80°C for 5 min, and held at 12°C in a thermocycler (Bio-Rad C1000 Thermal Cycler, Bio-Rad Laboratories, Hercules, CA). Reaction mixtures without DNA template and DNA from verified clean potato tubers obtained from the International Potato Center (CIP) healthy control lines served as negative controls. All reactions were visually assessed. Amplification products were confirmed by gel electrophoresis in 1.0% agarose gel in 1× TAE (10mM Tris HCl and 10mM EDTA) pH 8.0 at 120 V. The gel was stained using Gel Red at a final concentration of 0.5 mg/ml and the LAMP amplicons visualized under ultraviolet light. The LAMP reaction was performed using three technical replications.

*Sensitivity of* IpxC *LAMP and qPCR assays*. A pure culture of *R. solanacearum* phylotype IIB, strain UW551, was grown on semi-selective agar (Elphinstone et al. 1996) for 24 h at 30°C, and genomic DNA was extracted using a commercial kit (PureLink Genomic DNA Mini Kit). DNA extract was serially diluted 10-fold six times to 0.05 pg/µl, with a further twofold dilution to 2.5 pg/µl. DNA preparations from three independent dilution series were used to evaluate the sensitivity of the primers for qPCR and *IpxC* LAMP. The dilution series was used to determine the minimum concentration of *R. solanacearum* DNAthat could be detected by the *IpxC* LAMP assay and generate a standard curve to determine the concentration of *R. solanacearum* DNA in field samples.

IpxC *LAMP assay field validation*. For sample collection and preparation, potato tubers were randomly collected from 47 farmers’ fields in a zigzag fashion from 1/8th of a hectare in the major potato growing regions of Kenya (Meru, Nyeri, Kirinyaga, Limuru, Nyandarua, Koibatek, Molo, Uasin Gishu, Mt. Elgon, Kisii, Bomet, and Narok). The survey involved pooling 25 potato tubers per field to constitute a single sample. The pooled potato tubers were surface sterilized with 70% ethanol and aseptically cored at the stolon end (~0.1 g) using a potato coring device (courtesy of J. Smith, Fera Science Ltd., York, U.K.). The coring device was sterilized in 1% bleach and 70% ethanol and flamed between pooled potato samples to avoid crosscontamination between samples. The cored potato tubers were placed in a collection tube containing 3.75 ml of extraction buffer (phosphate buffer pH 7.0; Na_2_HPO_4_, KH_2_PO_4_, and antioxidant [tetrazolium pyrophosphate]). The mixture was transferred to a maceration bag and homogenized with hand tissue homogenizer (Bioreba AG, Reinach, Switzerland), and the supernatant was used for bacterialDNAextraction.

For DNA preparation, a boiling method (Weller et al. 2000) and a commercial DNA extraction kit (ZR Plant/Seed DNA MiniPrep) based on the manufacturer’s instructions were tested to evaluate DNA extraction methods that could produce high-quality DNA for LAMP assays. The boiling method (Weller et al. 2000) with modifications involved macerating a sample of 25 tuber stolon-end vascular tissue cores in 3.75 ml of extraction buffer (phosphate buffer pH 7.0; Na_2_HPO_4_, KH_2_PO_4_, and antioxidant [tetrazolium pyrophosphate]) in sterile maceration bag and inoculating at room temperature without shaking for 30 min. The resultant supernatant was transferred to a fresh centrifuge tube and a second centrifugation step performed at 180 × *g* for 10 min. The resulting pellet was suspended in 1 ml of molecular-grade water and 10,000 × *g* for 10 min. Approximately 800 ml of the supernatant was discarded. The remaining 200 ml was heated at 96°C for 4 min. Precipitation of the genomic DNA was done using 3 M sodium acetate mixed with 100% ethanol (1:1 vol. ratio), chilling at –20°C for 30 min, and centrifuging at 10,000 × *g* for 10 min. Final washing was done twice with 70% ethanol and subsequent centrifugation for 5 min. The DNA pellet was then air dried, dissolved in molecular-grade water, and incubated at 37°C for 5 min before use. The DNA extracted from boiling was tested using the LAMP assay to assess whether DNA was amplifiable using the method, whereas the DNA extracted using the commercial kit (ZR Plant/Seed DNA MiniPrep) was tested using qPCR and conventional PCR methods.

**Comparative evaluation of diagnostic tests.** Four detection assays (*IpxC* LAMP, qPCR, conventional PCR, and ELISA) were evaluated using field-collected potato tubers (Table 3). A control consisting of one infected tuber in 24 “disease-free” tubers was tested, in order to determine if a single infected tuber in a pool of healthy tubers could be consistently detected.

**Table 3:** Detection of *Ralstonia solanacearum* using nitrocellulose membrane enzyme-linked immunosorbent assay (NCM ELISA), polymerase chain reaction (PCR), loop-mediated isothermal amplification (LAMP), and quantitative PCR (qPCR) on field-collected potato tubers in Kenya^a^

Origin	Sample ID	NCM ELISA	Conventional PCR	IpxC LAMP (Thermocycler)	qPCR Mean Ct values 6 SD)^b^
Kirinyaga County	Kirinyaga-1	+	+	+	28.3 ± 0.1
	Kirinyaga-2	+	+	+	29.0 ± 0.4
	Kirinyaga-3	+	+	+	27.8 ± 0.3
	Kirinyaga-4	+	+	+	29.4 ± 0.7
Molo County	Molo-1	−	−	−	39.0 ± 0.2
	Molo-2	+	+	+	26.9 ± 0.1
	Molo-3	+	+	+	30.0 ± 0.2
	Molo-4	−	+	+	28.7 ± 0.3
Meru County	Meru-1	+	+	+	31.6 ± 0.4
	Meru-2	−	−	−	38.7 ± 0.3
	Meru-3	−	−	−	38.8 ± 0.4
	Meru-4	−	+	+	32.8 ± 0.2
Nyandarua County	Nyandarua-1	−	−	+	30.9 ± 0.4
	Nyandarua-2	+	+	+	28.4 ± 0.3
	Nyandarua-3	+	+	+	28.4 ± 0.1
	Nyandarua-4	+	+	+	31.7 ± 2.2
Nakuru County	Nakuru-1	−	−	−	ND
	Nakuru-2	+	+	+	28.1 ± 0.2
	Nakuru-3	−	−	−	37.9 ± 1.2
Nyeri County	Nyeri-1	+	+	+	34.7 ± 0.4
	Nyeri-2	−	+	+	34.6 ± 0.9
	Nyeri-3	+	+	+	33.6 ± 1.2
	Nyeri-4	+	+	+	32.3 ± 0.4
Narok County	Narok-1	−	+	+	34.4 ± 1.4
	Narok-2	+	+	+	27.1 ± 0.6
	Narok-3	+	+	+	29.1 ± 0.4
	Narok-4	+	+	+	25.5 ± 0.5
Bomet County	Bomet-1	+	+	+	26.2 ± 0.6
	Bomet-2	+	+	+	25.4 ± 0.1
	Bomet-3	−	+	+	33.6 ± 1.2
	Bomet-4	+	+	+	24.5 ± 0.4
Koibatek County	Koibatek-1	+	+	+	26.6 ± 0.8
	Koibatek-2	+	+	+	26.5 ± 1.1
	Koibatek-3	−	−	+	29.9 ± 0.1
	Koibatek-4	+	+	+	29.5 ± 1.6
Bungoma County	Elgon-1	+	+	+	25.3 ± 0.3
	Elgon-2	+	+	+	25.4 ± 0.3
	Elgon-3	+	+	+	23.1 ± 0.4
	Elgon-4	+	+	+	24.9 ± 0.7
Uasin Gishu County	UasinGishu-1	+	+	+	24.2 ± 0.5
	UasinGishu-2	+	+	+	25.6 ± 0.4
	UasinGishu-3	−	+	+	25.2 ± 0.2
	UasinGishu-4	+	+	+	25.1 ± 0.5
Kisii County	Kisii-1	+	+	+	26.5 ± 0.2
	Kisii-2	+	+	+	23.9 ± 0.0
	Kisii-3	+	+	+	23.8 ± 0.1
	Kisii-4	+	+	+	22.9 ± 0.4
Controls	Healthy potato tubers	−	−	−	ND
	No-template control	−	−	−	ND
	Positive control	+	+	+	15.8 ± 0.1

a Data from different detection techniques performed in three technical replications. The numbers in the sample ID (1 to 4) indicate the number of the fields tested in a region. A plus sign (+) indicates a positive test result for field potato samples tested (for NCM ELISA, purple color comparable to positive control; for conventional PCR [759/760], observed presence of amplified PCR products of 280 bp when viewed by gel electrophoresis; and for IpxC LAMP, translucent blue color). A minus sign (–) indicates a negative test result for field potato samples tested (for NCM ELISA, no color development on NCM ELISA nitrocellulose membrane; for conventional PCR [759/760], no amplified PCR product on gel; and for IpxC LAMP, translucent violet color). ND indicates not detected.

b Values are mean cycle threshold (Ct) of three replicates ± standard deviation for the 47 field samples tested using qPCR.

IpxC *LAMP*. LAMP reactions were performed as described above in a C1000 Touch thermocycler (Bio-Rad) using DNA extracted by boiling or the commercial kit. A plasmid positive control containing a 500-bp region of the *IpxC* gene was cloned using the TA cloning kit (Life Technologies, Carlsbad, CA) following manufacturer’s instructions. This clone (pMTx) contained the 150-bp target region of the *IpxC* LAMP primers. The plasmid positive control was tested independently in all LAMP reactions for field-collected samples.

*qPCR*. Previously published *R. solanacearum* qPCR primers RSF/ RSR targeting the *IpxC* gene (Table 1) (Chen et al. 2010) were used for comparison with *IpxC* LAMP. Amplification was performed in an ABI 7900HT Fast-Time PCR system (Applied Biosystems, Foster City, CA) in a 20-µl reaction containing 10 µl of SYBR Premix (Life Technologies), 2 µl (10 to 20 ng/µl) of template, and 0.4 ml of both forward and reverse primers (10 mM each). Reactions were performed according to the Chen et al. (2010) protocol with the following parameters: initial preheat for 10 min at 95°C, 40 cycles at 95°C for 20 s, 62°C for 25 s, 72°C for 35 s, and 85°C for 3 s. Fluorescence was detected at 85°C (Chen et al. 2010). All reactions were replicated three times.

Conventional PCR. PCR was performed in a thermocycler (Bio-Rad C1000 Thermal Cycler, Bio-Rad) as described by Opina et al. (1997) using the Rs-specific PCR primers 759/760 (Table 1). Briefly, PCR was carried out in 20-µl reactions containing PCR premix (Bioneer Corporation, Daejeon, Korea), 4 pmol of each primer, 7 µl of water, and 2 µl (10 to 20 ng/µl) of DNA. Reactions were heated to 96°C for 5 min and then cycled through 30 cycles of 94°C for 15 s, 59°C for 30 s, and 72°C for 30 s, followed by a final extension period of 10 min at 72°C. Samples (5 µl) of amplification products were resolved by gel electrophoresis in 2% agarose gel in 1× TAE (10mM Tris HCl and 10mM EDTA) pH 8.0 at 100 V. The gel was stained using Gel Red at a final concentration of 0.5 µg/ml and the PCR amplicons visualized under ultraviolet light.

*ELISA and strip test.* Pooled samples of 25 tubers were cored and macerated in extraction buffer containing 0.1 M citrate buffer (pH 5.6). Potato tuber samples were homogenized, and 500 µl of the tuber extract was mixed with 500 µl of broth in a 1.5-ml tube and incubated for 48 h with constant agitation (100 rpm). Dot blotting and ELISA were performed according to the CIP NCM ELISA kit for *R. solanacearum* (CIP, Lima, Peru). Positive samples were purple on nitrocellulose, whereas negative samples appeared cream to brown color. A few pooled potato tubers (15 field samples) were also tested using immunological strips (Bioreba) according to the manufacturer’s instructions (data not available).

**Data analysis.** To evaluate the sensitivity and consistency of the detection assays, mean score and standard deviation among technical and biological replicates were calculated using Microsoft Excel.

## Results

**Fig. 1:**
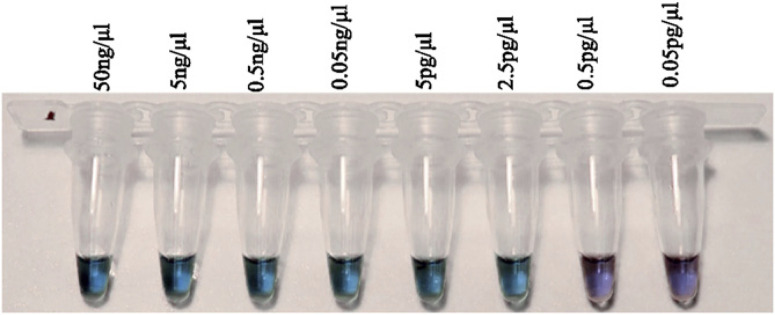
Analytical sensitivity of *IpxC* loop-mediated isothermal amplification to detect DNA of *Ralstonia solanacearum* (translucent blue indicates positive reactions, and translucent violet indicates negative reactions). A dilution series 10-fold of *R. solanacearum* total DNA, phylotype IIB, strain UW551.

**Fig. 2:**
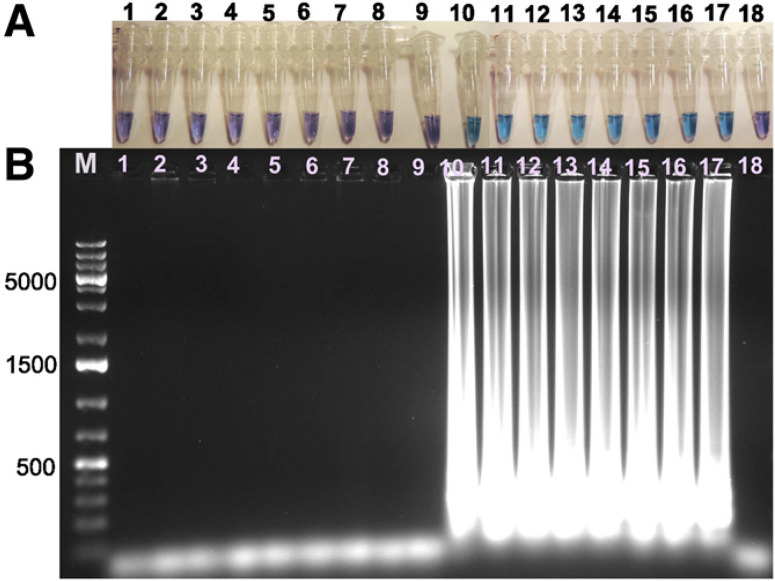
Specificity of *IpxC* loop-mediated isothermal amplification following incubation at 65°C for 1 h. **A**, Hydroxynaphthol blue visualization reaction, and **B**, gel electrophoresis. Non-*R. solanacearum* species complex: lane M, 100 kb Plus ladder; 1, CU3351; 2, 71; 3, ATCC 15713; 4, CG49; 5, 0651; 6, 09110; 7, 0430; 8, 361; and 9, 83-01-08. *Ralstonia* species complex strains: 10, J; 11, UW551; 12, GMI1000; 13, CMR15; 14, PSI07; 15, ACH0732; 16, ATCC 11696; and 17, ATCC BAA-1115. Lane 18 is potato tubers.

LAMP assay development. Specificity of IpxC LAMP primers. After a preliminary evaluation of the 10 newly-designed primer sets for sensitivity and specificity to R. solanacearum and other soilassociated bacteria, the IpxC primer set was selected for further analyses (Table 1). Our LAMP primer sets that targeted egl, fliC, and 16S rRNA were either not specific to R. solanacearum or were not as sensitive as the IpxC primers when assayed with hydroxynaphthol blue (data not shown). The IpxC primers produced translucent bright blue color changes that could easily be differentiated from the translucent light purple negative reactions (Fig. 1). The IpxC primers consistently amplified DNA from all the 11 R. solanacearum species complex strains tested, including *R. syzygii* subsp. *indonesiensis*, except for *R. syzygii* subsp. celebesensis, also known as blood disease bacteria (BDB) strain currently classified in phylotype IV. None of the soil-borne bacteria produced a product based on color changes (translucent violet for negative test, translucent blue for positive test) with the hydroxynaphthol and confirmed by gel electrophoresis (ladderlike band for positive test, no ladder-like band for negative test) (Fig. 2). IpxC LAMP and qPCR detected R. solanacearum DNA concentrations between 50 ng/µl and 2.5 pg/µl (mean cycle threshold [Ct] values 22 to 36) (Table 4).

**Table 4:** *Ralstonia solanacearum* dilution series of phylotype IIB, strain UW551, detected by quantitative polymerase chain reaction (qPCR) assay using RSF/RSR and by IpxC LAMP primer reactions^a^

Detection method	Parameter				DNA concentrations			
		50 ng/µl	5 ng/µl	0.5 ng/µl	0.05 ng/µl	5 pg/µl	2.5 pg/µl	0.5 pg/µl
qPCR, primer RSF/RSR	Ct values	22.3	25.5	27.8	31.3	34.5	35.8	>36.0
	SD	0.3	0.4	0.5	0.4	0.5	1.3	NC
*IpxC* LAMP	Hydroxynaphthol blue	+	+	+	+	+	+	−

a The primer set RSF/RSR was reported by Chen et al. (2010). Ct = mean cycle threshold for three independent serial dilutions; SD = standard deviation for three independent serial dilutions; NC = not calculated; + = positive test result; and − = negative test result.

Field validation of IpxC LAMP assay. IpxC LAMP with field samples. Diseased potatoes were collected from 12 counties in Kenya. R. solanacearum was detected in 42 of 47 field samples (Table 3). All 24 out of 47 infected pooled tuber samples from both extraction methods (Table 3) tested in a thermocycler gave consistent results for positive and negative samples. A translucent blue color for positive and a translucent violet color for negative were observed for samples extracted using both the boiling and commercial kit (Fig. 1). Negative controls containing healthy potato tuber DNA and nuclease-free water resulted in no amplification. In addition, the plasmid positive control and a separate control (one infected tuber in 24 disease-free tubers) tested positive for R. solanacearum.

*qPCR.* The standard deviation of three independent technical replicates was <1 for samples within the linear range (Ct value less than 36), except for the last twofold dilution, when the standard deviation was 1.28. Of the total 47 samples, 42 samples that tested positive with *IpxC* LAMP also tested positive with qPCR within the linear range of the standard curve with Ct values of 23 to 35 (Table 3). *R. solanacearum* DNA was not detected consistently above the linear range of 36 (data not shown). Consequently, all samples above the Ct value of 36 were assessed as negative. No amplification signals were detected in healthy potato extracts and the no-template control.

*Conventional PCR*. Conventional PCR assay (Opina et al. 1997) amplified *R. solanacearum* DNA in 40 infected pooled tubers out of 47 pooled tuber samples (Table 3). Primers 759/760 (Opina et al. 1997) amplified PCR products of 280 bp when viewed by gel electrophoresis (data not shown). Two of the seven samples (Koibatek-3 and Nyandarua-1) that tested negative by conventional PCR were positive when tested by qPCR (Ct values of 30 and 31) and *IpxC* LAMP.

*ELISA*. NCM ELISA detected 34 out of 47 pooled potato samples tested (Table 3). Of the total 47 pooled tuber samples, eight samples tested positive using qPCR (Ct values between 25 and 35) and LAMP but were negative by ELISA. Positive samples were purple on NCM ELISA, whereas negative samples appeared cream to brown color. The same results were obtained for the 15 samples tested with the strip test (Bioreba), thereby demonstrating consistency in the test results of the two methods, except for one sample (UasinGishu-3), which was negative by NCM ELISA and positive by strip test (data not shown).

## Discussion

Potato farming in Kenya is endangered by diseases such as bacterial wilt disease, which cannot be detected through field inspection, thus requiring laboratory testing. However, seed potato certification and testing in Kenya have not fully embraced nucleic acid-based testing methods, with current practices still centered on antibody-based technologies such as ELISA. Although ELISA remains the popular tool in most labs in Kenya, it is acknowledged as having some cross-reactivity (Priou et al. 1997), resulting in false positives, which are costly to the grower and make it less suitable for seed certification. Increased sensitivity of the ELISA kit can be achieved by inclusion of an enrichment step through culturing of the test sample in semiselective broth for 48 h prior to detection. The current standard practice involves collecting and transporting many tubers back to the diagnostic facility for ELISA testing, which could be an expensive venture with a tedious and cumbersome process. Coring of tubers in the field can improve efficiency in sampling because only tuber cores are taken back to the lab for detection as long as testing is undertaken within 48 h (Okiro et al. 2016).

Current nucleic acid-based methods for detection of *R. solanacearum* are considered the most effective (Cellier et al. 2015; Dreo et al. 2014; Nikitin et al. 2018; Stulberg et al. 2016). However, they employ the use of sophisticated equipment that may be out of reach of many small labs in Kenya, coupled with the high cost of reagents especially for qPCR and the need to employ highly qualified personnel (Dreo et al. 2014; Lenarčič et al. 2014; Nikitin et al. 2018; Stulberg et al. 2016). An important advantage with a LAMP assay over PCR and immunoassays is the ability to amplify DNA at a constant temperature of 60 to 65°C without the need of an expensive thermocycler. The detection limit is equivalent or higher than PCR with a shorter detection time (Goto et al. 2009). The assay provides a simple visual discrimination of test results by eye without specialized equipment. A colorimetric LAMP assay requires no analysis of amplification product after incubation, minimizing cases of contamination and time required for analysis. This makes LAMP ideal for routine testing and seed certification. However, the hydroxynaphthol blue color change can be perceived variably by different observers (Wastling et al. 2010).

The *IpxC* LAMP assay reported here has adequate sensitivity and specificity and is relatively fast; thus, it can be useful in highthroughput testing (Goto et al. 2009). The *IpxC* LAMP assay detected DNA of *R. solanacearum* directly from potato tubers, eliminating the cumbersome and time-consuming culturing processes. A DNA extraction method based on boiling the sample makes the assay affordable. The *IpxC* LAMP assay utilized a low-technology LAMP colorimetric endpoint detection identified by visual inspection; thus, no specialized equipment was required to interpret results, increasing its versatility for adoption.

The sensitivity of *IpxC* LAMP was comparable to that of qPCR (2.5 pg/ml) with a level of sensitivity suitable for reliable confirmation of the presence of *R. solanacearum* in latent and symptomatic potato tubers. To confirm the sensitivity of the LAMP and qPCR assays, a dilution below the detection limit (0.5 pg/µl) was included and tested, which indicated that the positive reactions for each of the dilutions were not false, and all the reactions were replicated three times. All the test methods evaluated in this study were able to detect a pooled sample containing one infected tuber in 24 disease-free potato tubers, demonstrating the overall sensitivity of all the assays with a negative control giving no amplification. The *IpxC* LAMP primers amplified DNA from all the 11 *R. solanacearum* strains tested except for *R. syzygii* subsp. *celebesensis* (BDB) and none of the other potato and soil bacteria (Fig. 2).

The *IpxC* LAMP reaction time took 1 h, which was comparable to qPCR. Conventional PCR had a reaction time of 1 h 40 min with additional time for gel electrophoresis, making *IpxC* LAMP and qPCR the quickest methods of testing compared with conventional PCR and ELISA. The 48-h incubation step of the ELISA method aimed at mitigating the lower sensitivity resulted in qPCR, conventional PCR, and LAMP being 48 h quicker than ELISA (Priou et al. 1997).

Developing diagnostic tools that can be used by diagnostic facilities without extensive resources is in high demand and necessary for agricultural support services. LAMP is an appropriate diagnostic tool for resource-limited settings, and its simple visualization format is a straightforward method of detection. The colorimetric assay described here has been previously reported to have superior qualities than the existing colorimetric assays (Wastling et al. 2010). In addition, the LAMP assay was compared with existing nucleic-and antibody-based methods and was as reliable as other existing nucleic-based assays. This method is sensitive, rapid, and inexpensive, making it suitable for work in labs with limited facilities, and it has the potential to be adopted for seed potato certification purposes.

## References

[R1] Cellier G., Moreau A., Chabirand A., Hostachy B., Ailloud F., and Prior P. 2015 A duplex PCR assay for the detection of Ralstonia solanacearum phylotype II strains in Musa spp. PLoS One 10:e0122182. 25811378 10.1371/journal.pone.0122182 PMC4374791

[R2] Chen W. P., and Kuo T. T. 1993 A simple and rapid method for the preparation of gram-negative bacterial genomic DNA. Nucleic Acids Res. 21:2260. 8502576 10.1093/nar/21.9.2260 PMC309503

[R3] Chen Y., Zhang W., Xin L., Ma Z.,Bo L., Allen C., and Jian-Hua G. 2010 A real-time PCR assay for the quantitative detection of Ralstonia solanacearum in horticultural soil and plant tissues. J. Microbiol. Biotechnol. 20:193-201. 20134252

[R4] Demo P., Lemaga B., Kakuhenzire R., Shultz S., Borus D., Baker I., Woldegiorgis G., Parker M. L., and Shulte-Geldermann E. 2015 Strategies to improve seed potato quality and supply in Sub-Saharan Africa: Experience from interventions in five countries. Pages 155-167 In: Potato and Sweetpotato in Africa: Transforming the Value Chains for Food and Nutrition Security. Low J, Nyongesa M, Quinn S, and Parker M, eds. CAB International, Wallingford, U.K.

[R5] Dittapongpitch V., and Surat S. 2003 Detection of Ralstonia solanacearum in soil and weeds from commercial tomato fields using immunocapture and the polymerase chain reaction. Phytopathology 151:239-246.

[R6] Dreo T., Pirc M., Ramsak Z., Pavsic J., Milavec M., Zel J., and Gruden K. 2014 Optimising droplet digital PCR analysis approaches for detection and quantification of bacteria: A case study of fire blight and potato brown rot. Anal. Bioanal. Chem. 406:6513-6528. 25173868 10.1007/s00216-014-8084-1

[R7] Elphinstone J. G., Hennessy J., Wilson J. K., and Stead D. E. 1996 Sensitivity of different methods for the detection of Pseudomonas solanacearum in potato tuber extracts. EPPO/OEPP Bull. 26:663-678.

[R8] Gildemacher P. R., Kaguongo W., Ortiz O., Tesfaye A., Woldegiorgis G., Wagoire W. W., Kakuhenzire R., Kinyae M., Nyongesa M., Struik P. C., and Leeuwis C. 2009 Improving potato production in Kenya, Uganda and Ethiopia: A system diagnosis. Am. J. Potato Res. 52:173-205.

[R9] Gosch C., Gottsberger R. A., Karl S., and Fischer T. C. 2012 Blue EaLAMP—A specific and sensitive method for visual detection of genomic Erwinia amylovora DNA. Eur. J. Plant Pathol. 134: .

[R10] Goto M., Honda E., Ogura A., Nomoto A., and Hanaki K. 2009 Colorimetric detection of loop-mediated isothermal amplification reaction by using hydroxy naphthol blue. Biotechniques 46:167-172. 19317660 10.2144/000113072

[R11] Guidot A., Elbaz M., Carr`ere S., Siri M. I., Pianzzola M. J., Prior P., and Boucher C. 2009 Specific genes from the potato brown rot strains of Ralstonia solanacearum and their potential use for strain detection. Phytopathology 99:1105-1112. 19671014 10.1094/PHYTO-99-9-1105

[R12] Hayward A. C. 1991 Biology and epidemiology of bacterial wilt caused by Pseudomonas solanacearum. Annu. Rev. Phytopathol. 29:65-87. 18479193 10.1146/annurev.py.29.090191.000433

[R13] Huang J., Wu J., Li C., Xiao C., and Wang G. 2009 Specific and sensitive detection of Ralstonia solanacearum in soil with quantitative, real-time PCR assays. J. Appl. Microbiol. 107:1729-1739. 19486215 10.1111/j.1365-2672.2009.04364.x

[R14] Kubota R., and Jenkins D. M. 2015 Real-time duplex applications of loopmediated amplification (LAMP) by assimilating probes. Int. J. Mol. Sci. 16: 4786-4799. 25741765 10.3390/ijms16034786 PMC4394449

[R15] Kubota R., Vine B. G., Alvarez A. M., and Jenkins D. M. 2008 Detection of Ralstonia solanacearum by loop-mediated isothermal amplification. Phytopathology 98:1045-1051. 18943743 10.1094/PHYTO-98-9-1045

[R16] Lenarčič R., Morisset D., Pirc M., Llop P., Ravnikar M., and Dreo T. 2014 Loop-mediated isothermal amplification of specific endoglucanase gene sequence for detection of the bacterial wilt pathogen Ralstonia solanacearum. PLoS One 9:e96027. 24763488 10.1371/journal.pone.0096027 PMC3999105

[R17] Mitarai S., Okumura M., Toyota E., Yoshiyama T., Aono A., Sejimo A., Azuma Y., Sugahara K., Nagasawa T., Nagayama N., Yamane A., Yano R., Kokuto H., Morimoto K., Ueyama M., Kubota M., Yi R., Ogata H., Kudoh S., and Mori T. 2011 Evaluation of a simple loop-mediated isothermal amplification test kit for the diagnosis of tuberculosis. Int. J. Tuberc. Lung Dis. 15:1211-1217. 21943848 10.5588/ijtld.10.0629

[R18] Mori Y., Nagamine K., Tomita N., and Notomi T. 2001 Detection of loopmediated isothermal amplification reaction by turbidity derived from magnesium pyrophosphate formation. Biochem. Biophys. Res. Commun. 289:150-154. 10.1006/bbrc.2001.5921 11708792

[R19] Murata H., Fons M., Chatterjee A., Collmer A., and Chatterjee A. K. 1990 Characterization of transposon insertion out-mutants of Erwinia carotovora subsp. carotovora defective in enzyme export and of a DNA segment that complements out mutations in E. carotovora subsp. carotovora, E. carotovora subsp. atroseptica, and Erwinia chrysanthemi. J. Bacteriol. 172: 2970-2978. 2160934 10.1128/jb.172.6.2970-2978.1990 PMC209096

[R20] N’Guessan C. A., Abo K., Fondio L., Chiroleu F., Lebeau A., Poussier S., Wicker E., and Koné D. 2012 So near and yet so far: The specific case of Ralstonia solanacearum populations from Cˆote d’ Ivoire in Africa. Phytopathology 102:733-740. 22533876 10.1094/PHYTO-11-11-0300

[R21] Nikitin M. M., Statsyuk N. V., Frantsuzov P. A., Dzhavakhiya V. G., and Golikov A. G. 2018 Matrix approach to the simultaneous detection of multiple potato pathogens by real-time PCR. J. Appl. Microbiol. 124:797-809. 10.1111/jam.13686 29297963

[R22] Notomi T., Okayama H., Masubuchi H., Yonekawa T., Watanabe K., Amino N., and Hase T. 2000 Loop-mediated isothermal amplification of DNA. Nucleic Acids Res. 28:e63. 10871386 10.1093/nar/28.12.e63 PMC102748

[R23] Obura E., Masiga D., Wachira F., Gurja B., and Khan Z. R. 2011 Detection of phytoplasma by loop-mediated isothermal amplification of DNA (LAMP). J. Microbiol. Methods 84:312-316. 21185882 10.1016/j.mimet.2010.12.011

[R24] Okiro L. A., Nyanjom S. G., and Parker M. L. 2016 Coring method of sampling potato tubers to detect Ralstonia solanacearum. Int. J. Exp. Agric. 14:1-6.

[R25] Ophel K., and Kerr A. 1990 Agrobacterium vitis sp. nov. for strains of Agrobacterium biovar 3 from grapevines. Int. J. Syst. Bacteriol. 40:236-241.

[R26] Opina N., Tavner F., Hollway G., Wang J. F., Li T. H., Maghirang R., Fegan M., Hayward A. C., Krishnapillai V., Hong W. F., Holloway B. W., and Timmis J. N. 1997 A novel method for development of species and strainspecific DNA probes and PCR primers for identifying Burkholderia solanacearum (formerly Pseudomonas solanacearum). Asia Pac. J. Mol. Biol. Biotechnol. 5:19-30.

[R27] Priou S., Gutarra L., and Aley P. 2006 An improved enrichment broth for the sensitive detection of Ralstonia solanacearum (biovars 1 and 2A) in soil using DAS-ELISA. J. Plant Pathol. 55:36-45.

[R28] Priou S., Gutarra L., Fernandez H., and Aley P. 1997 Sensitive detection of Ralstonia solanacearum in latently infected potato tubers and soil by postenrichment ELISA. Pages 111-121 In: Impact on a Changing World: Program Report, 1997-98. International Potato Center, Lima, Peru.

[R29] Ravindran A., Levy J., Pierson E., and Gross D. C. 2012 Development of a loop-mediated isothermal amplification procedure as a sensitive and rapid method for detection of ‘Candidatus Liberibacter solanacearum’ in potatoes and psyllids. Phytopathology 102:899-907. 22881872 10.1094/PHYTO-03-12-0055-R

[R30] Stulberg M. J., Rascoe J., Li W., Yan Z., Nakhla M. K., and Huang Q. 2016 Development and comparison of TaqMan-based real-time PCR assays for detection and differentiation of Ralstonia solanacearum strains. Curr. Microbiol. 73:542-549. 27402488 10.1007/s00284-016-1091-z

[R31] Stulberg M. J., Shao J., and Huang Q. 2015 A multiplex PCR assay to detect and differentiate select agent strains of Ralstonia solanacearum. Plant Dis. 99: 333-341. 30699705 10.1094/PDIS-05-14-0483-RE

[R32] Tomita N., Mori Y., Kanda H., and Notomi T. 2008 Loop-mediated isothermal amplification (LAMP) of gene sequences and simple visual detection of products. Nat. Protoc. 3:877-882. 18451795 10.1038/nprot.2008.57

[R33] Tomlinson J. A., Barker I., and Boonham N. 2007 Faster, simpler, more-specific methods for improved molecular detection of Phytophthora ramorum in the field. Appl. Environ. Microbiol. 73:4040-4047. 17449689 10.1128/AEM.00161-07 PMC1932743

[R34] Tomlinson J. A., Boonham N., and Dickinson M. 2010a Development and evaluation of a one-hour DNA extraction and loop-mediated isothermal amplification assay for rapid detection of phytoplasmas. J. Plant Pathol. 59: 465-471.

[R35] Tomlinson J. A., Dickinson M. J., and Boonham N. 2010b Rapid detection of Phytophthora ramorum and P. kernoviae by two-minute DNA extraction followed by isothermal amplification and amplicon detection by generic lateral flow device. Phytopathology 100:143-149. 20055648 10.1094/PHYTO-100-2-0143

[R36] Tomlinson J. A., Ostoja-Starzewska S., Adams I. P., Miano D.W., Abidrabo P., Kinyua Z., Alicai T., Dickinson M. J., Peters D., Boonham N., and Smith J. 2013 Loop-mediated isothermal amplification for rapid detection of the causal agents of cassava brown streak disease. J. Virol. Methods 191:148-154. 22820076 10.1016/j.jviromet.2012.07.015

[R37] Wagura A. G., Wagai S. O., Manguro L., and Gichimu B. M. 2011 Effects of selected plants’ extracts on in vitro Ralstonia solanacearum (Smith), the causal agent of bacterial wilt of Irish potatoes. Asian J. Plant Pathol. 10:66-72.

[R38] Wastling S. L., Picozzi K., Kakembo A. S. L., and Welburn S. C. 2010 LAMP for human African trypanosomiasis: A comparative study of detection formats. PLoS Negl. Trop. Dis. 4:e865. 10.1371/journal.pntd.0000865 PMC2970543 21072228

[R39] Weller S. A., Elphinstone J. G., Smith N. C., Boonham N., and Stead D. E. 2000 Detection of Ralstonia solanacearum strains with a quantitative, multiplex, real-time, fluorogenic PCR (TaqMan) assay. Appl. Environ. Microbiol. 66:2853-2858. 10877778 10.1128/aem.66.7.2853-2858.2000 PMC92083

[R40] Yasuhara-Bell J., Kubota R., Jenkins D. M., and Alvarez A. M. 2013 Loopmediated amplification of the Clavibacter michiganensis subsp. michiganensis micA gene is highly specific. Phytopathology 103:1220-1226. 23802869 10.1094/PHYTO-03-13-0078-R

